# Empirical evidence that diversionary feeding increases productivity in ground-nesting birds

**DOI:** 10.1098/rspb.2024.2921

**Published:** 2025-06-25

**Authors:** Jack Anthony Bamber, Chris Sutherland, Kenny Kortland, Xavier Lambin

**Affiliations:** ^1^ School of Biological Sciences, University of Aberdeen, Aberdeen AB24 2TZ, UK; ^2^ Centre for Research into Ecological and Environmental Modelling, University of St Andrews, St Andrews KY16 9LZ, UK; ^3^ Forestry and Land Scotland, Inverness IV3 8NW, UK

**Keywords:** capercaillie, conservation conflict, diversionary feeding, empirical, ground-nesting birds, predation, predator-prey, productivity

## Abstract

The recovery of predator populations may negatively impact other species of conservation concern, leading to conservation conflicts. Evidence-based solutions are needed to resolve such conflicts. Robust, large-scale field experiments provide the most rigorous evidence to justify new forms of intervention. Still, they are hard to implement and often call for indirect and non-invasive monitoring. In this study, we used camera traps to experimentally evaluate diversionary feeding to reduce conservation conflict and non-invasively monitor capercaillie hen productivity over 3 years under a randomized control (unfed) and treatment (fed) design. Diversionary feeding significantly increased the probability that a detected hen would have a brood. Brood size decreased over time, but the change did not differ between fed and unfed treatments. Importantly, the increased chance of having a brood with diversionary feeding substantially increases overall productivity at the end of the sampling season. This was just 0.82 (0.35–1.29) chicks per hen without diversionary feeding, and more than doubled to 1.90 (1.24–2.55) with diversionary feeding. This study provides compelling empirical evidence that diversionary feeding positively affects productivity, offering an effective non-lethal solution to the increasingly common conservation conflict where both predator and prey are afforded protection.

## 1. Introduction

Controversies around predator control have become acute in the context of recovering predators, where predatory animals that were once reduced in number or eradicated are now returning to historical numbers and distributions through conservation actions [1]. Robust evaluation of management strategies is vital to support decision-making in conservation management, ensuring interventions suit the current context [2]. As such, historically successful management interventions may become less suitable with changes in management goals and acceptance of certain methodologies, including where species assemblages are reforming under legal protection and conservation efforts [3,4]. Nevertheless, many traditional management interventions are still used despite little evidence of their suitability and, in some instances, despite evidence that they are ineffective under current conditions. Lethal control of predators to maintain or produce a harvestable surplus of prey species and to protect livestock remains a widely used management practice [5]. In its current form, lethal predator control delivers only short-term reductions in abundance owing to compensatory immigration and breeding, requiring repeated disruption to predator communities [6,7]. Protection of some predator species also reduces the efficacy of lethal control because changes in predation rates by the non-controlled predators can also be compensatory [8]. Moreover, social norms increasingly challenge the legitimacy of lethal control, whether for the maintenance of game species or for the protection of species of conservation concern [9]. The lack of long-term efficacy, moral objections and the differing management practices across landownerships contribute to a growing pressure to find alternatives to lethal control. Yet, barriers exist to the uptake of alternatives, such as the attachment to traditional practices and a lack of reliable or trusted evidence on their efficacy and practicality [10].

Predator recovery can be controversial if it increases predation by recovering predators on prey species that are declining and of conservation concern or valued (game and farming interest) [1,11]. Some examples of these conflicts are recovering grey wolves (*Canis lupus*) predating Finnish forest reindeer (*Rangifer tarandus fennicus*) [12] and the predation pressure presented by a range of meso-carnivores consuming the nests and chicks of ground-nesting birds [13,14]. In such circumstances, relieving predation pressure on prey populations may be necessary to restore productivity and allow prey population persistence [15]. The legal protection of some predators also prevents population control unless there is evidence that culling predators under licence would achieve specific management objectives. This is another area of uncertainty, as direct evidence is rarely available. This increases the polarization between proponents of traditional lethal control and conservationists unwilling to sacrifice recent conservation gains with the resumption of culling [11]. However, there remains uncertainty about the circumstances under which non-lethal interventions offer a feasible solution to these conflicts.

One promising non-lethal intervention to reduce predation impact is diversionary feeding, the deliberate provision of alternative food to divert predation pressure away from species of management interest [16,17]. It is predicated on the premise that opportunistic predators will preferentially consume readily accessible, provisioned food rather than searching for cryptic prey [18]. Historical applications of diversionary feeding aimed at moving large ungulates away from train lines or bears (Ursidae) away from human settlements have had mixed successes [19,20]. Diversionary feeding has been demonstrated more clearly to reduce predation on both game and threatened bird species by various predators (e.g. red foxes (*Vulpes vulpes*) on black grouse (*Lyrurus tetrix*), red kites (*Milvus milvus*) on red grouse (*Lagopus scotica*) and kestrels (*Falco tinnunculus*) on little terns (*Sternula albifrons*) [21–23]). However, the extent to which the foraging behaviour of problematic predators can be modified through management is unknown in most specific contexts. Accordingly, strong evidence for the effects of diversionary feeding, as well as consideration of the potential positive (reduced predation) and negative (numerical and aggregation responses) effects, is necessary before it is deployed as an alternative to traditional lethal control.

The conservation conflict between the legally protected and recovering pine marten (*Martes martes*) [24] and the drastically declining capercaillie (*Tetrao urogallus*) [25] is a good example of a context where diversionary feeding holds promise. Pine martens are seasonal nest predators implicated in the continued decline of ground-nesting capercaillie [26,27]. A randomized experiment recently demonstrated an 83% reduction in depredation rates on artificial nests by pine marten with short-term diversionary feeding [17]. The use of artificial nests as a proxy for nest predation allowed experimental rigour but fell short of providing direct evidence that diversionary feeding does reduce predation on actual capercaillie nests. In this study, we directly address this knowledge gap using a landscape-scale feeding experiment to quantify the effects of diversionary feeding on capercaillie productivity—measured as the expected number of chicks per hen. Through non-invasive monitoring of capercaillie broods over time, we quantify to what extent diversionary feeding reduces the impact of predation on capercaillie breeding success.

## 2. Methods

### (a) Study area

This study was conducted within the Cairngorms Connect landscape, a 600 km^2^ multi-stakeholder ecological restoration project in the Cairngorms National Park, Scotland (57°09′47.5″N 3°42′47.0″W). The area is within the stronghold of Scotland’s largest remaining capercaillie populations [28] and hosts a recovering guild of generalist predators, including pine marten, red fox and badger (*Meles meles*) as the main terrestrial predators, alongside carrion crow (*Corvus corone*), common buzzard (*Buteo buteo*) and ten scarcer raptor species. No lethal predator control was conducted during sampling, and fox control was phased out in the preceding 3 years. A vital aspect of managing this landscape is culling red and roe deer (*Cervidae*), using copper ammunition, for forest regeneration. This provides a potential year-round subsidy to predators and an easily accessible food source for diversionary feeding. Pilot monitoring of deer carrion during culling revealed that all generalist predators actively consumed carrion. Partner organizations sought non-lethal ways to manage predator impacts without resorting to population control, granting permission for experimentation. This experiment allowed the deployment and assessment of diversionary feeding as an emergency intervention, with the option to halt if adverse effects were observed.

### (b) Diversionary feeding experimental design

The experiment was performed in a control (unfed) and treatment (fed) design. Unfed and fed sampling grid cells were deployed randomly across the experiment [17]. The grid cells used in this study were a subset of grid cells from Bamber *et al.* [17] with recent records of capercaillie presence (historical brood counts, capercaillie sightings and lek activity from 2018 onwards) to provide an increased chance of capercaillie detection.

Grid cells were 1 km^2^ in size, separated by a 1 km^2^ spacing grid cell to maximize the independence of the treatment. The size of sampling units was chosen to encompass the typical daily movement range of a pine marten, the focal nest predator (49 ha in females and 54 ha in males [29]). This size also encompassed the average home range of capercaillie broods, 0.4 km^2^ [30], making it unlikely that broods would use multiple grid cells during experimentation. Fed and unfed grid cells were switched each year across three years of sampling (2021−2023) to reduce any influence of grid cell properties that was not accounted for through the initial randomization of grid cell selection, with unfed grid cells becoming fed in the following year and *vice versa*.

Diversionary feeding stations were deployed in 2021, 2022 and 2023, from the last week of April to the first week in July, encompassing capercaillie egg laying, incubation and the first 30 days after hatching. Approximately, 10 kg of deer carrion was placed at each feeding station and restocked every two weeks to ensure fresh food was always present for any consumers (ungulate carrion is known to be a nutrient-rich and appealing resource scavenged by the focal meso-carnivores of this study [31]). Any meat remaining when restocking was left in place to build scent cues to increase detection by local predators. Stations were deployed within approximately 100 m of the centre of the fed grid cells, avoiding water courses by >20 m and human tracks and trails by >50 m. The flipping of treatments between years meant that grid cells had the same treatment in 2021 and 2023, but feeding stations were at least 50 m from the previous location. The short feeding timeframe and changing of feeding locations across years aimed to reduce the risk of increasing population size or causing long-term spatial aggregation of predators, respectively. Feeding stations monitored by camera traps detected feeding by all predators present in the system at differing rates across the study years and locations. For full details of the core experimental design and feeding station usage, see Bamber *et al.* [17].

### (c) Brood monitoring through camera trapping

To detect capercaillie non-invasively, camera traps (Browning Recon Force Advantage and Recon Force Elite; set at 3-shot burst with 5 s intervals between) were deployed on dust baths (microsites known to be used by capercaillie broods [32]). Non-invasive monitoring was required owing to capercaillie’s schedule 1 protected status, prohibiting any disturbance during breeding without licensing [33]. Further, partner organizations did not want the study to deploy invasive monitoring methods (i.e. radio tracking of broods or dog counts) out of concern that they negatively impact capercaillie [34].

Camera deployments on dust baths occurred following ‘cold searches’ of grid cells. Thirty-three grid cells were initially identified as having historical capercaillie presence, making them suitable for cold search for camera deployments. Cold searches consisted of walking a transect within the sampling grid cell, scanning initially for capercaillie signs (feathers, droppings or sightings). If signs were found, this began localized searching of dusty areas (upturned root plates, exposed earth on hillsides or bare earth in standing tree roots). Searching dusty areas led to camera deployment if any signs of use, such as dust bowls, capercaillie droppings or capercaillie feathers, were detected within a dust bath. Cameras were deployed in 30 of the 33 historical capercaillie grid cells, with contemporary evidence of occupancy. Cold searches were performed every year alongside diversionary feeding, with some sites that initially had no suitable dust baths detected subsequently added when suitable dust bath sites were located (table 1). If several dust baths were in proximity to each other, those with hen feathers and fresh signs of recent use were selected for deployment. Cameras were deployed in clusters (at 2–4 dust baths) within grid cells to increase the probability of detection rate, with only one camera deployed per individual dust bath [35]. If more than four dust baths with signs of recent use were detected within a grid cell, dust baths farthest away from each other were selected for deployment. If no suitable dust bath with signs of use could be found, no camera was deployed.

**Table 1.** Camera deployments during sampling. Separated into individual cameras and unique, independent sampling grid cells (containing multiple cameras (2–4)). The number of cameras and grid cells, alongside the number of grid cells detecting a capercaillie hen (with or without a brood) and a capercaillie brood, are presented.

year	cameras with detections (day)				unique grid cells		
	cameras deployed	cameras with a detection	hen detections	hen with brood	grid cells with cameras deployed	with hen detection	hen with brood
2021	57	24	47	23	25	16	12
2022	87	24	57	36	28	16	13
2023	102	60	204	122	30	25	16
total	246	108	308	181	83	57	41

Monitoring of dust baths took place from late May to mid-September and included the most vulnerable period for recently hatched capercaillie, which hatch in early June, rely on their mothers in the first few weeks of life, and follow their mothers through to dispersal in early autumn [36].

### (d) Data extraction

Camera trap images were processed primarily using the Digicam and CamTrapR framework to tag and extract detections of capercaillie, tagging males, females and broods as three different types of detection [37]. Detections were separated by day for independence. The tagged images from 2021 and 2022 were used to train the Conservation AI software to automatically identify capercaillie cocks and hens to extract capercaillie images passively from 2023 [38]. We extracted encounters identified by conservation AI as containing capercaillie hens, and a manual assessment was performed on this subset of images using the same workflow as 2021 and 2022 to identify hen detections (removing false positives). Images were correctly identified to contain a capercaillie hen 95.4% of the time (13 532 correct, 14 119 identified). Brood counts were created manually from detections of capercaillie hens, assigning a chick count to each image during the encounter (all images detected in sequence from arrival at the dust bath till leaving). The chick count assigned to the individual encounter for analysis was the most chicks seen on an image in a sequence. This provided the response variable for modelling: the presence of a brood (0 or 1) and, if chicks were detected with a hen, the brood size (range: 1–8). Total detections per year and grid cell varied (table 1), with extremes, one grid cell detecting a hen capercaillie on 29 separate days and others having only one detection across the entire sampling period.

### (e) Statistical analysis

We modelled reproductive output using a two-part zero-truncated (or hurdle) Poisson regression model [39]. First, the binary outcome of brood presence (reproductive success; barren = 0, brood = 1) was modelled using binomial regression with a logit link to estimate the probability of a hen having a brood. Conditional on a brood being present, the number of chicks (brood size) was modelled using a truncated Poisson regression with a log link. The Poisson distribution was truncated at zero to reflect the biological constraint that chick counts are only observed for brooding hens, with the probability mass function truncated at 0 (i.e. does not include 0). By combining both models, estimates provide an intuitive estimate for the number of chicks per hen, a standard measure of bird productivity.

The binomial response and the conditional counts were modelled as a function of covariates using linear mixed modelling approaches. Given the experimental nature of our study, we considered two covariates as predictors for both processes. We included a binary treatment effect (fed/unfed) to explicitly test for the effect of diversionary feeding on both the probability of a hen being barren and brood size. We also included a time of year effect (ordinal day) to account for the potential for brood presence and brood size to vary (i.e. decrease) over the 10-week study period. Ordinal day ran from day 0, relating to 5 June, to day 98, relating to the 12 September, encompassing the period chicks are likely with hens. We included treatment–time interactions for both processes to test for treatment-specific temporal changes in the presence and number of chicks. To account for the non-independence of detections in the same sample grid cell (we deployed, on average, three cameras per grid cell), we used gridyear (grid cell location and year of sampling combined) as a random effect, resulting in 57 unique grid-years in total (23 fed, 34 unfed). Including the year within the random effect also controlled for interannual variation in productivity.

Considering all combinations of covariates resulted in a total of 25 models. Models were fitted in R [40] using the GLMMTMB function from the ‘glmmTMB’ package [41]. Model selection was conducted based on Akaike information criterion (AIC) model comparisons [42] produced from the ‘glmmTMB’ models, and predictions and 95% confidence intervals were calculated using the ‘ggeffects’ package [43].

The estimates from the combined model described above provided information that can be used to parameterize a deterministic population projection model to explore the importance of diversionary-feeding-induced changes in productivity on population growth. To do so, we adapted the parameters from the post-breeding capercaillie population projection model of Moss *et al*. [44], which was developed for the same population. We parameterized the breeding probability using productivity rate data from 13 tracked hens [36] using our estimates of productivity (number of chicks per hen) from fed and unfed treatments. Deterministic population growth rates and projections for populations with and without diversionary feeding were performed in R using the ‘popbio’ package [45].

## 3. Results

### (a) Brood analysis

**Figure 1.**
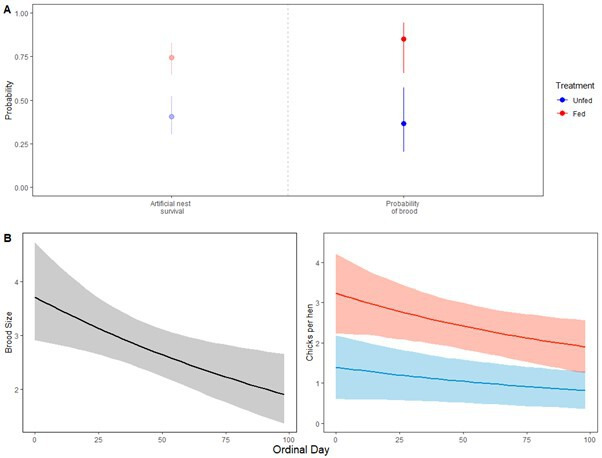
Impact of diversionary feeding on capercaillie productivity. (A) In the left panel, we show the probability of artificial nest survival over 28 days, as shown in the previous assessment of diversionary feeding [17]. After the dashed line, in the right panel, we show the predicted probability of detecting a hen with a brood (binomial model) in this study. (B) In the left panel, we show the predicted number of chicks (Poisson model) when a hen is detected with a brood, over the sampling season. In the right panel, we show the combined estimate (combined effects of the probability of having a brood (binomial model) and change over time on overall predicted productivity (chicks per hen, Poisson model)), under the different diversionary feeding treatments, presenting the estimated chicks per hen over time. Ordinal day 0 is 5 June. Estimates of predictions for treatment fed are shown in red, blue is for treatment unfed and black where there was no treatment effect. The 95% confidence intervals for all models are depicted in corresponding treatment colours.

The presence or absence of diversionary feeding affected the probability that a hen was detected with a brood. However, this probability did not change over time. The top supported model, using AIC, included the effect of treatment only on the probability of having no brood (the binomial ‘zeros’ model) and the effect of ordinal day only on the expected brood size (the truncated Poisson count model) (table 2). This means that, specifically, diversionary feeding significantly reduced the probability of a hen being barren when detected from 0.848 (95% CI: 0.66–0.94) when unfed to 0.37 (0.57–0.94) with feeding (estimate: −2.27, s.e.: 0.71) (figure 1A). Note that the binomial model estimates the probability of a 0 (barren), and 1 minus that probability is the probability of having a brood. We report this inverse value from here for clearer intuition (figure 1).

**Table 2.** Akaike information criterion (AIC) table to show a model selection of variables; the table contains the top four models, with ΔAIC <2, out of the possible 25 model combinations, across the two parts of the hurdle model, treatment and ordinal day.

description	AIC	ΔAIC	parameters
count: day of seasonzero: treatment	983.311	0	4
count: day of season zero: treatment & day of season (interaction)	984.614	1.303	6
count: day of season zero: treatment and day of season (additive)	984.718	1.408	5
count: day of season & treatment (additive) zero: treatment	985.137	1.826	5

In terms of inference about brood size, our results suggest that productivity declines throughout the study (−0.007, s.e.: 0.002), but is not affected by the presence of diversionary feeding (table 3). While the probability of having a brood was higher with diversionary feeding, the expected brood size for a hen with a brood is not different and the expected brood size declines over time at the same rate in fed and unfed treatments (figure 1B). Importantly, when the two parts of the modelling process are combined to include both the brood count and brood probability model to produce an estimate of chicks per hen, this results in a stark productivity difference between fed versus unfed grid cells. For example, by the end of sampling (ordinal day 98), the predicted number of chicks per hen is 1.90 (1.24–2.55) with diversionary feeding, which is 131% higher than in the unfed grid cells, which is 0.82 (0.35–1.29, figure 1B).

**Table 3.** Coefficients of zero truncated hurdle model showing changes in the probability of a barren hen being detected (zero) and the brood size change when a brood was detected (count). Intercept for count is day 0 (5 June), for zero treatment, unfed). Dashes indicate effects not present in the count or zero sub-models. Combined model, accounting for the count combined with zero probability.

	count (Poisson, log link)			zero (binomial, logit link)		
	estimate	s.e.	*p*	estimate	s.e.	*p*
intercept	1.311	0.124	<2 × 10^−16^	0.543	0.423	0.199
treatment: fed	—	—	—	−2.268	0.712	0.001
ordinal day	−0.007	0.002	0.007	—	—	—

### (b) Influence of productivity increase

Using our estimates of chicks per hen, we compared population growth rates in the presence and absence of diversionary feeding. In the unfed scenario, the growth rate was *λ* = 0.903, a population decline that is broadly consistent with predictions from Moss *et al.* [44]. Introducing diversionary feeding into the model increased the population growth rate to *λ* = 1.109. Thus, widescale adoption of diversionary feeding shifted deterministic population growth by 20%.

**Figure 2.**
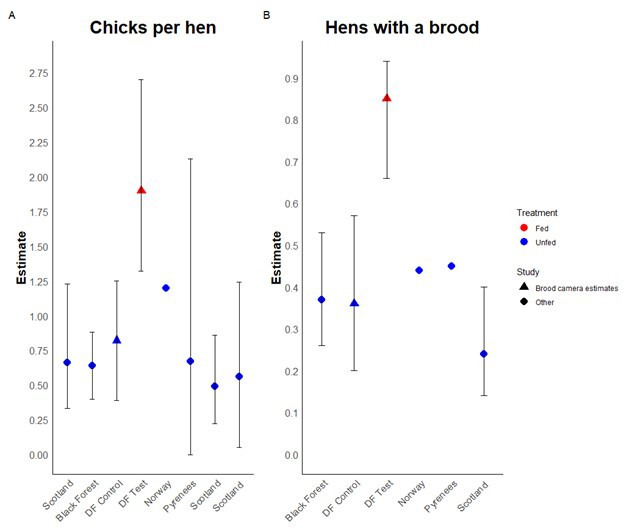
Collection of productivity estimates for capercaillie recruitment across Europe. (A) Chicks per hen, and (B) brooded hens. Studies from the literature are shown with diamonds, and estimates from this current study are shown with triangles, with diversionary feeding (DF) prediction in red. When available, the range of estimates is included as black error bars.

To further compare the estimates from this study and the impact of diversionary feeding with other estimates of capercaillie breeding success, we performed a literature search for studies published in the last 10 years, with multiple years of sampling, that estimated capercaillie productivity. The literature search highlighted seven studies [28,30,46–50] that had assessed capercaillie breeding output, with a calculation of either chicks per hen or the rate at which a hen was detected with a brood. A comparison of these studies with the results from the current diversionary feeding experiment shows two key results (figure 2). First, our baseline control estimates align closely with previous findings, giving confidence in the accuracy of the estimates produced from this non-invasive monitoring technique. Second, diversionary feeding raises productivity levels beyond those observed across the international capercaillie range, including areas where overall capercaillie populations are considered more stable than in Scotland.

## 4. Discussion

We found that the productivity of detected capercaillie in this study increases with the presence of diversionary feeding. In doing so, we demonstrated that the reduction in predation rates (by badgers and pine martens) observed in a field experiment using artificial nests as a proxy response variable seemingly translates to an increase in the detection of capercaillie with broods. This indicates the increased survival of actual capercaillie nests, and subsequent increases in productivity. Areas with diversionary feeding increased the predicted number of chicks per hen at the end of the dependence period by over 131%, from 0.82 to 1.9 chicks per hen. Our result affirms that nest depredation significantly contributes to breeding failure in ground-nesting capercaillie [50], and shows that diversionary feeding is a feasible, non-lethal, solution to this conservation conflict. Through strong inference afforded by a rare, randomized, replicated field experiment, there arguably is now ‘certainty of the evidence’ that diversionary feeding is demonstrably effective in reducing the impact of predation on capercaillie nesting success without undesirable harm as per the conservation evidence framework [51]. Accordingly, practitioners can now consider deploying this method with the knowledge that the barrier of lack of evidence has been addressed.

Bamber *et al.* [17] found that nest survival increased to 74% with diversionary feeding, compared with 40% without it, mainly owing to a significant decrease in depredation by pine martens and, to a lesser extent, badgers. Legitimate concerns remained that the use of artificial nests, including differences in predator behaviour between natural and artificial nests and the influence of scent cues (both increasing or decreasing predation), may have caused spurious results [52,53]. For instance, few artificial nests (1.3%) were consumed by crows and foxes, despite previous evidence of capercaillie nest depredation by these species [54,55] but mixed evidence on the actual rates of corvids [54]. The alignment between the probabilities of detecting a hen with a brood and of artificial nest survival (figure 1) alleviates these concerns. Thus, it appears from our results that diversionary feeding effectively reduces (mostly badger and pine marten) predation of capercaillie nests, obviating the need for lethal population control of these protected species. By implication, the quantitative contribution of crows and foxes must be small. Diversionary feeding is a viable tool to reconcile predator recovery with the seemingly conflicting conservation goals of protecting capercaillie.

We found no positive effect of our experiment on either brood size or its decay rate over time, indicating that diversionary feeding does not reduce chick predation. While predation likely contributes to chick loss, the guild of predators responsible is likely wider and includes raptors, for example goshawks, which predate capercaillies and chicks [56,57]. Goshawks were only detected once across 75 diversionary feeding deployments, meaning they are unlikely to be influenced by diversionary feeding. Brood size declined significantly throughout the season, with predictions from the count model showing a decline of 51% (from 3.7 to 1.9 chicks) over 98 days. Multiple factors have been linked to chick deaths in capercaillie, outside of predation, most notably cold and wet springs, through their impact on insect prey availability [50,58]. Although diversionary feeding was insufficient to improve chick survival, interventions that improve habitat may be more suitable to achieve this, such as increasing blaeberry understorey through burning, grazing and mowing [59].

Diversionary feeding more than doubled the predicted productivity to 1.9 chicks per hen. Diversionary feeding achieves this by increasing the number of broods rather than influencing brood size. This far surpasses the 0.6–1.1 chicks per hen previously suggested as necessary for population stabilization in Scotland [44]. Projecting population growth with the increase in chicks per hen linked to diversionary feeding shifted the annual population growth rate from a 9% decline to a 10% increase. While crude, this exercise helps demonstrate the potential and promise of diversionary feeding on the population growth of a species that many have suggested is all but extinct [25]. Our key message is that diversionary feeding can substantially mitigate the impact of nest predation on population growth, consistent with the prediction that nest loss more than chick loss impacts productivity [50].

### (a) Wider management implications

Predicted chicks per hen more than doubled when the impact of nest predation was reduced by diversionary feeding. This improved productivity matches seen in capercaillie populations during peak productivity, often linked to peak vole years when depredation is deemed lowest owing to abundant alternative prey, with our study taking place during both low and increasing phases in the rodent cycle [17,60] (figure 2). Our evidence indicates that similarly high productivity rates could be realized by managing the impact of predation in other parts of the southern range of capercaillie (Cantabria, Alps, Bavaria), where predation is also deemed to contribute to rapid population declines [26,61]. Our evidence shows that diversionary feeding effectively mitigates the impact of multiple nest predators, providing a tool for land managers wishing to intervene effectively on predator impact.

A barrier to action has been the concern raised by practitioners that feeding predators will increase overall predator abundance or cause local aggregation [16]. The feeding protocol we tested involved a short, eight-week-long seasonal feeding pulse that matched egg laying and incubation of capercaillie. This was short enough to minimize any impact on predator survival and to avoid a numerical response. The lack of a negative impact of diversionary feeding on brood size in this study may indicate that there is no numerical response associated with feeding by predators. Notably, Olejarz & Podgórski [62], found no consistent effect of supplementary feeding on the home ranges of terrestrial mammals, with feeding choices likely impacting at an individual level and not at a population level, implying that uniform aggregation is unlikely with mammalian predators. However, impacts on raptor communities may differ. Managers considering diversionary feeding should not let a fear of aggregation prevent an investigation into the method under their specific contexts, but it is an area requiring further study.

Managing the impact of increased predation by generalist predators that have recovered from past persecution and now thrive in anthropogenic landscapes is a global issue in conservation [1,63]. Substantial resources are expended in managing predation to rescue an ever-growing list of at-risk ground-nesting birds, such as waders and grouse, from decline [14,64–66]. In many cases, elevated predator abundances are symptoms of modified ecosystems rather than the ultimate cause of the decline [67], meaning that population control could negatively impact native predators without any long-term benefits to prey at the cost of broader conservation due to these conservation conflicts [68]. Our evidence shows significant potential for impact-based intervention to alleviate unwanted predation and indicates the viability of coexistence conservation strategies within predator conflicts.

## Data Availability

Data has been archived on Zenodo [69].
